# Enhanced Domain Adaptation for Foot Ulcer Segmentation Through Mixing Self-Trained Weak Labels

**DOI:** 10.1007/s10278-024-01193-9

**Published:** 2024-07-17

**Authors:** David Jozef Hresko, Peter Drotar, Quoc Cuong Ngo, Dinesh Kant Kumar

**Affiliations:** 1 IISLab, Technical University of Kosice, Letna 1/9, Kosice, 04200 Kosicky Kraj Slovakia; 2 School of Engineering, RMIT University, 80/445 Swanston St, Melbourne, 3000 VIC Australia

**Keywords:** Medical image segmentation, Self-training, Mixup augmentation, Weak label, Domain adaptation, Diabetic foot ulcer, Semi-supervised learning

## Abstract

Wound management requires the measurement of the wound parameters such as its shape and area. However, computerized analysis of the wound suffers the challenge of inexact segmentation of the wound images due to limited or inaccurate labels. It is a common scenario that the source domain provides an abundance of labeled data, while the target domain provides only limited labels. To overcome this, we propose a novel approach that combines self-training learning and mixup augmentation. The neural network is trained on the source domain to generate weak labels on the target domain via the self-training process. In the second stage, generated labels are mixed up with labels from the source domain to retrain the neural network and enhance generalization across diverse datasets. The efficacy of our approach was evaluated using the DFUC 2022, FUSeg, and RMIT datasets, demonstrating substantial improvements in segmentation accuracy and robustness across different data distributions. Specifically, in single-domain experiments, segmentation on the DFUC 2022 dataset scored a dice score of 0.711, while the score on the FUSeg dataset achieved 0.859. For domain adaptation, when these datasets were used as target datasets, the dice scores were 0.714 for DFUC 2022 and 0.561 for FUSeg.

## Introduction

One common complication associated with diabetes is diabetic foot ulcers (DFU) which occur with about 25% of people with diabetes [1]. Proper treatment leads to the healing of the majority of DFU within 12 weeks, however, some of the ulcers do not heal within this period [2] and can become chronic. Delayed treatment of chronic DFU can result in amputations; a quarter of patients with chronic DFU undergo limb amputation within 6–18 months after the initial assessment, with a 5-year post-amputation mortality rate as high as 50% [3]. Thus, early detection of DFU that are chronic is important.

The assessment of the DFU is based on the shape and area of the ulcer, with the area of the ulcer expected to reduce by half in the fourth week of presentation [4]. For computerized analysis, this requires the image of the DFU to be accurately segmented, which however is not trivial due to the differences in skin texture and color, the shape of the ulcer, and variations in the lighting conditions. This can make the traditional image segmentation techniques unsuitable.

The use of neural networks for segmentation of medical images has been proposed for several applications. Convolutional Neural Networks (CNNs) have successfully been used in the segmentation of medical images such as lung X-ray [5], CT scans of abdominal area [6] and even in the area of diabetes [7]. However, these networks require large, well-labeled datasets. However, medical image datasets with meticulously labeled instances across diverse patient conditions is a time-consuming and resource-intensive process and thus are typically small and often unbalanced [8]. Researches have been developing methods to overcome this problem by developing methods suitable for noisy and imprecise, i.e., weakly labeled, images [9–11].

Self-training is a simple yet effective alternative paradigm to handle weakly labeled data and generally belongs to the group of semi-supervised learning algorithms [12]. The model is initially trained on a small set of accurately labeled data. Subsequently, the model is employed to predict labels for unlabeled or partially labeled instances in the dataset. These predicted labels are then added to the training set, expanding the labeled data pool. The model is iteratively retrained on this augmented dataset, refining its predictions and gradually improving its performance. This process continues through multiple iterations, with the model progressively self-improving and refining its segmentation capabilities.

While self-training methods have shown the potential, but may introduce noise and errors in real-life conditions, impacting the model’s accuracy [13]. Domain adaptation complements self-training by addressing the challenges associated with the domain shift between the source and target datasets. By aligning feature distributions across different domains, domain adaptation methods enhance the model’s ability to generalize effectively, even in the presence of diverse imaging conditions [14]. This interplay between self-training and domain adaptation is particularly valuable in scenarios where comprehensive labeled datasets are scarce, as it allows the model to leverage both its own predictions and domain-informed adaptations to improve segmentation accuracy in real-world clinical settings.

Motivated by the successful use of self-training for synthetic label generation, and for weak label segmentation, we propose an improved self-training segmentation method. While this still relies on the concept of iterative retraining of neural network, but with the incorporation of generated labels by mixup augmentation [15] into the training process. We argue that while the plain incorporation of synthetic labels or noisy human-crafted labels limits the network capabilities to capture the required attributes, our approach will be without any misleading external factors. We evaluated the proposed method for the task of semantic segmentation of diabetic foot ulcers. The results demonstrate that mixup augmentation can utilize weak labels more effectively and increase the robustness and precision of the neural network in medical image segmentation when unseen (new) data are presented.

The rest of the paper is organized as follows. The “Related Work” section describes the related works, and identifies the common problems related to domain adaptation and utilization of weak labels in segmentation problems. It focuses on recent improvements and solutions utilizing neural networks to mitigate mentioned problems in both researched topics. In the following sections, we describe the proposed method followed by the description of diabetic foot ulcer datasets used in our experiments. The next section presents Results followed by Discussions where the external validation, potential applications, limitations, possible improvements, and future direction are discussed.

## Related Work

Application of self-training in deep learning architectures is considered to be a way of the utilization of pseudo-labeling principles, which relies on iterative training with a limited amount of labels, resulting in progressive fine-tuning of the model itself by the prediction on the unlabeled part of the dataset [16].

Researchers have focused on the refinement of pseudo-labels to reduce initial noise, which is a common problem [17, 18]. The early works used conditional random fields to produce pseudo-labels and utilize them in an effective way [19]. However, the naive assumption that the majority of the produced segmentations are correct can lead to incorrect training and classification of targeted pixels. More recent papers have focused on the improvement of methods, which used thresholds and various filters to differentiate between quality samples and weak ones [20, 21].

The alternative is to focus on the improvement of the generation of pseudo-labels instead of selecting quality ones. One good example of these methods is FixMatch, which shifts its interest toward the quality of the generated samples. However, this still implies quality thresholding to be the efficient way [22]. FixMatch utilized consistency regularization to ensure uniform predictions on augmented unlabeled data and employed confidence thresholding to discard less reliable predictions. This dual approach offers both, computational efficiency and ease of implementation. An interesting idea of label exploration to boost quality was presented in Chang et al. [23]. It identifies sub-categories within semantic classes, generates pseudo-labels based on these sub-categories, and iteratively refines them during training. By leveraging sub-category information, the method improves the quality of pseudo-labels and enhances segmentation performance without requiring pixel-level annotations.

More novel approaches shift toward the idea of utilization of class activation maps (CAM) to produce refined annotations. For example, [24] used this approach to extract the feature pixels of presented classes with a combination of the class labels to learn another fully connected layer with softmax cross-entropy loss. Once converged, reactivated CAM is used to produce high-quality refined masks. Similar concepts related to CAM were proposed in Wu et al. [25], where authors explored the collaborative information based on the homogeneity of discriminative activation maps to utilize supervision information of image-level labels. Song et al. [26] proposed a framework named Fully-CAM that applies the features from all convolution layers to obtain complete discriminative regions. In the context of weakly-supervised semantic segmentation, Chen et al. [27] reactivated traditionally used binary cross-entropy loss in converged CAM by using additional softmax cross-entropy loss to produce masks with a smaller ambiguity.

While all of the mentioned works showed promising results, it is still arguable how these methods can adapt to more diverse medical domains, especially in scenarios where data availability is extremely scarce and label presence is rare. To explore these scenarios, we focused on the task of image segmentation in the domain of diabetic foot ulcers (DFU). Our goal here was to improve the utilization of labels, which are often few and also lack quality. In the current paper, we presented an alternative approach to mitigate the scarcity of quality labels in the context of domain adaptation via mixing self-trained labels. We did not use any CAM methods or other generative concepts to produce synthetic pseudo-labels from the target domain. Instead, in our approach we demonstrated that utilization of mixup augmentation can boost domain adaptation capabilities without exhaustive modifications of the self-training concept. We focused on the improvement of the core concept of the self-training method to enhance domain adaption in the not so well-explored medical domain of diabetic foot ulcers.

## Datasets

To evaluate the domain adaptation capabilities of the proposed method, we selected two distinct datasets containing images of foot ulcers that were used to develop and test the models. The best one was then validated on a third completely unseen dataset. The following subsections describe the three datasets.

### DFUC 2022 Dataset

Diabetic Foot Ulcers (DFUC) 2022 dataset [28, 29] contains 2000 fully annotated images of diabetic foot ulcers captured under various medical conditions. We utilized only publicly available annotated data from this dataset. The dataset received approval from the UK National Health Service Research Ethics Committee (Ref. No. 15/NW/053). We utilized only publicly available annotated data from this dataset. During the stage of data gathering three different cameras were used, namely Kodak DX4530, Nikon D3300, and Nikon COOLPIX P100. Close-ups of the entire foot, positioned around 30–40 cm away from the ulcer plane with a parallel orientation, were acquired. Instead of a camera flash, the authors preserved consistent colors in the images using only room lights. The original size of images (ranging from 1600x1200 to 3648x2736 pixels) was down-sampled to 640x480, preserving the aspect ratio. The ground truth labels of the ulcer areas were produced by a podiatrist and a consultant physician specializing in diabetic foot care, each with over five years of professional experience. In cases of disagreement, a third specialist podiatrist reviewed the image.

### FUSeg Dataset

The Foot Ulcer Segmentation (FUSeg) dataset is a deidentified dataset with patient consent and ethics documents waived by the institutional review board of the University of Wisconsin-Milwaukee [30]. This was created with the collaboration of Advancing the Zenith of Healthcare (AZH) Wound and Vascular Center. It originally contains 1210 foot ulcer images collected over two years from 889 patients, out of which 986 were fully annotated and were used in our experiments. The original images were captured using both a Canon SX 620 HS digital camera and an iPad Pro, in environments with unregulated lighting conditions and diverse backgrounds. Captured images were resized to 512x512 pixels. Initial labels underwent subsequent scrutiny and adjustments by wound care specialists with over 20 years of experience in wound care, along with medical assistants from a collaborating wound clinic who monitored the patients. In instances of complexity or disagreement, the labeling team collectively decided the labels. Throughout the labeling process, tissues such as granulation, slough, and eschar were marked and annotated as wounds.

### RMIT Dataset

To evaluate the generalization capability of the proposed approach on one more dataset we included also RMIT. This dataset has been collected in outpatient clinics by clinicians independent of the researchers. The dataset has not been annotated yet.

In a prospective longitudinal observational study conducted at Austin Health, Melbourne, Australia, inpatients and outpatients aged 30 and above with both type I and II diabetes who sought treatment for DFUs were recruited. The study has been reported earlier in Aliahmad et al. [31]. The data collection protocol was approved by the Austin Health Human Ethics Committee (Ref. No. LNR/15/Austin/51), and enrolled 104 diabetic patients, excluding 25 due to insufficient data, or pre-existing conditions. Wagner grading was used to categorize ulcers, considering only grades I and II plantar ulcers and excluding ischaemic, post-amputation, and complex ulcers. Only plantar ulcers were considered, and heel and dorsal DFUs were excluded. The images were taken using a Nikon DSLR in the DFU clinic after debridement and washing of the wound. The images were resized to 512x512 pixels to maintain the aspect ratio. Since this dataset has not been labeled yet, we provide only visualization of segmentation and no quantitative results.

## Methodology

Domain adaptation addresses the challenge of applying a model trained on a source domain such as medical images from one set of imaging devices or protocols to a target domain such as images from a different set of devices, protocols, or patient populations, where the data distribution may differ significantly. This discrepancy in data distribution can severely degrade the performance of segmentation models due to their reliance on large amounts of labeled data that are often costly or impractical to obtain for every new domain.

Domain adaptation techniques aim to bridge this gap by enabling models to learn domain-invariant features, or by transforming the target domain data to more closely resemble the source domain, thereby improving the model’s ability to generalize across different domains without the need for extensive labeled data in the target domain. This problem can be defined as follows. Given a source domain $D_S = \{(x_i^s, y_i^s)\}_{i=1}^{N_s}$ with $N_S$ labeled samples where $x_i^s$ is an input image and $y_i^s$ is its corresponding segmentation mask, and a target domain $D_T = \{(x_j^t)\}_{j=1}^{N_t}$ with $N_t$ unlabeled samples, the goal of domain adaptation is to learn a predictive function $F: X -> Y$ that performs well on $D_T$, despite the difference in data distributions $P_S(X,Y) \ne P_T(X,Y)$.

### Self-Training

Self-training is a semi-supervised learning technique where a model iteratively improves its performance by utilizing its own predictions to generate pseudo-labels for unlabeled data. The process begins with training the initial model on a labeled source dataset. Once the model is trained, it is used to predict labels for unlabeled data, effectively creating weak labels or pseudo-labels. These pseudo-labeled data are then incorporated into the training set, augmenting the original labeled data. The model is then retrained on this expanded dataset, which includes both the original labeled data and the newly pseudo-labeled data. This process is repeated iteratively until convergence or a predefined stopping criterion is met. The advantage of self-training is that it allows for the utilization of large amounts of unlabeled data, which are often abundant and cheaper to acquire compared to labeled data, thereby increasing data utilization and potentially improving model generalization. Additionally, the iterative nature of self-training enables the model to progressively refine its predictions and learn from its own mistakes, potentially leading to better performance over time. One major concern is the propagation of errors: if the initial model produces inaccurate predictions, these errors can propagate and be amplified throughout the training process, leading to degraded performance or convergence to sub-optimal solutions. Moreover, pseudo-labels generated by the model may be uncertain or incorrect, particularly for challenging or ambiguous cases, potentially hindering rather than improving model performance.

### Mixup of Self-Trained Weak Labels in Ulcer Segmentation

We propose the introduction of mix-up augmentation to mitigate some of the limitations associated with self-training in ulcer segmentation. Mixup augmentation is a data augmentation technique where new samples are created by linearly interpolating pairs of existing samples and their labels.

By blending images and their corresponding labels, mixup augmentation creates smoother decision boundaries, which helps the model become more resilient to noisy or incorrect pseudo-labels generated during self-training. Consequently, the risk of overfitting to erroneous predictions is mitigated. Secondly, mixup augmentation aids in improving generalization by encouraging the model to learn more meaningful representations of the data distribution. By generating synthetic samples along the interpolation path between two existing samples, the technique guides the model to capture the underlying structure of the data more effectively. This reduces the likelihood of overfitting to specific instances or biases introduced by pseudo-labels. By leveraging mixup augmentation alongside self-training, the model can effectively utilize both labeled and unlabeled data, thereby improving performance while minimizing the risk of overfitting to noisy or uncertain pseudo-labels.

**Fig. 1:**
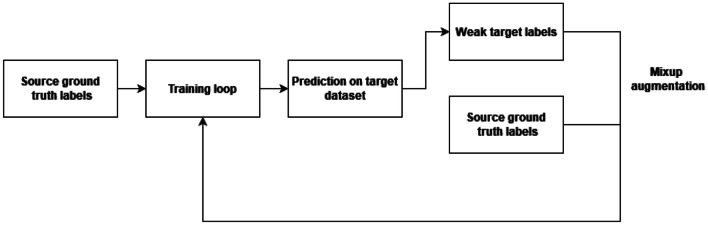
Overview of proposed method

An overview of the proposed method is shown in Fig. 1.

In the proposed approach, we start with the usual training process. A model *M* is trained on a source dataset $D_S = \{(x_i^s, y_i^s)\}_{i=1}^{N_s}$, where each $x_i^s$ is an input image and $y_i^s$ is its corresponding ground truth segmentation mask, by minimizing a loss function $\mathcal {L}_{src}$, typically a segmentation loss such as cross-entropy or Dice loss:

1 $$\begin{aligned} \mathcal {L}_{src}(M) = \frac{1}{N_s} \sum _{i=1}^{N_s} \ell (M(x_i^s), y_i^s). \end{aligned}$$

Trained model *M* is used to perform predictions on the target dataset $D_T = \{(x_j^t)\}_{j=1}^{N_t}$ to generate weak labels $\tilde{y}_j^t = M(x_j^t)$ for each image in the target dataset.

In the second step, the mixup technique is applied to create new training examples by linearly combining images and their labels from two datasets. For a pair of images, $x_a$,$x_b$ and their labels $y_a$,$y_b$, the mixup generates a new image-label pair $(x',y')$ as follows:

2 $$\begin{aligned} x' = \lambda x_a + (1 - \lambda ) x_b, \quad y' = \lambda y_a + (1 - \lambda ) y_b \end{aligned}$$

where $\lambda$ is distributed according Beta distribution $\lambda \sim \beta (\alpha , \alpha )$, where $\alpha \in (0,\infty )$.

**Fig. 2:**
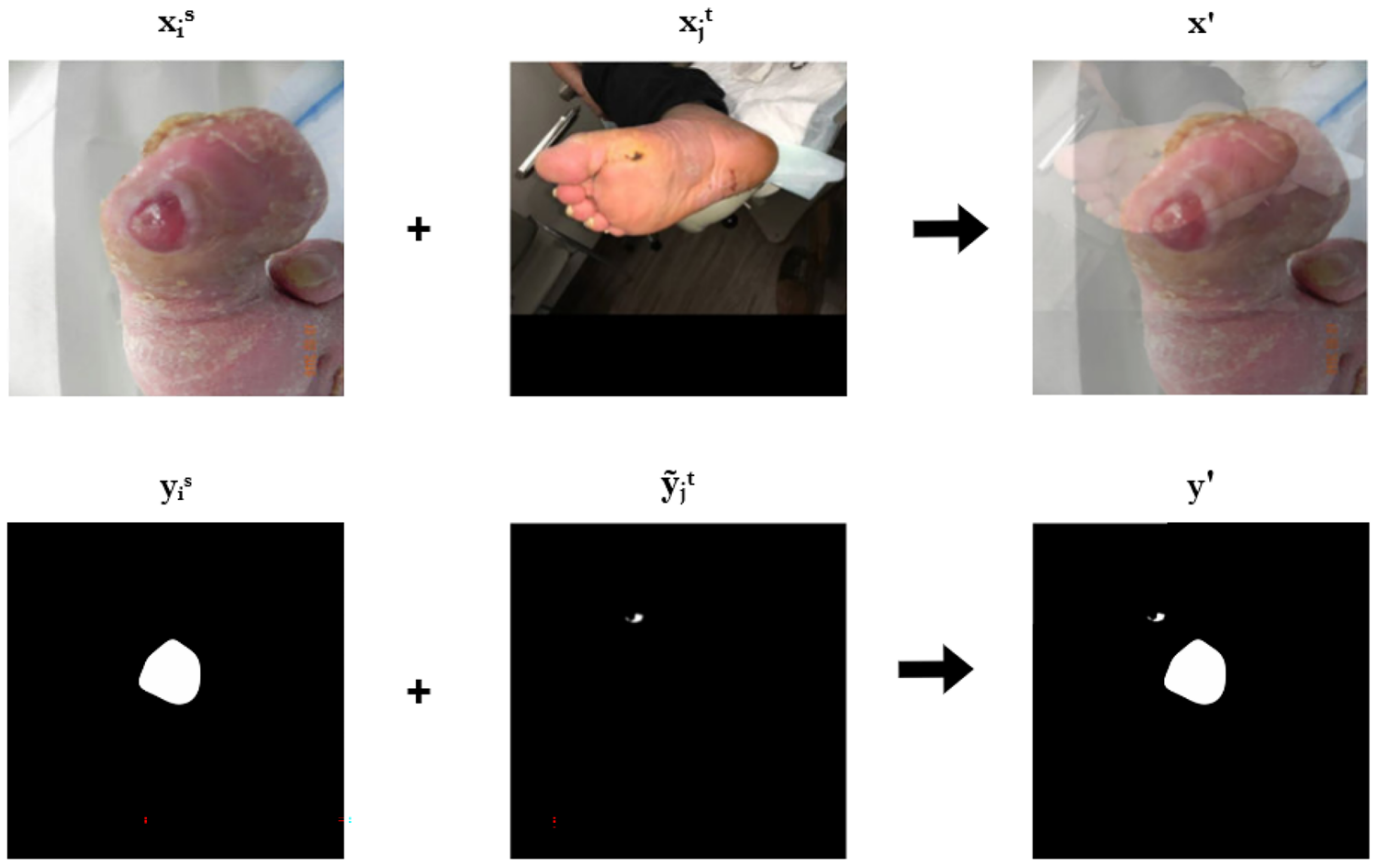
Example mix-up augmentation of source and self-trained target sample

In our approach, mixup is applied to the source and weakly labeled target data to create a mixed dataset $D_{mix}$ by applying mixup between the source dataset ground truth labels $y_i^s$ and the weak labels $\tilde{y}_j^t$. An example of this mixup is depicted in Fig. 2. The resulting dataset is then defined as

3 $$\begin{aligned} D_{mix} = \{(\lambda x_i^s + (1 - \lambda ) x_j^t, \lambda y_i^s + (1 - \lambda ) \tilde{y}_j^t)\}_{i,j}. \end{aligned}$$

The objective then becomes to train (or fine-tune) the model *M* on this mixed dataset $D_{mix}$ by minimizing the adapted loss function $\mathcal {L}_{mix}$, which is defined over the mixup-generated examples:

4 $$\begin{aligned} \mathcal {L}_{mix}(M) = \frac{1}{|D_{mix}|} \sum _{(x', y') \in D_{mix}} \ell (M(x'), y'). \end{aligned}$$

The model is optimized by minimizing $\mathcal {L}_{mix}$, thereby encouraging it to learn from both the precise ground truth labels in the source domain and the approximate, weak labels in the target domain, adjusted by the mixup strategy to encourage domain invariance and regularization:

5 $$\begin{aligned} M^* = \arg \min _M \mathcal {L}_{mix}(M). \end{aligned}$$

This process can be repeated multiple times as needed. In our experiments, we found that a single loop was sufficient to observe improvements over the standard self-training approach. We believe that well-known benefits and properties of mixup augmentation such as improved generalization and smoothing of decision boundaries, can improve the quality of weak labels by mixing correctly labeled data, thus reducing noise, and introducing certainty and already learned attributes of targeted class. Furthermore, this approach preserves the original self-training method, without requiring complex modifications to the existing architectures and hardware requirements.

## Results

The proposed method was used to delineate the foot ulcers and the results were compared with the manual ground truth annotations. We used two medical datasets to train and test the models: DFUC 2022 and FUSeg, and the third was used to validate this model. Sørensen-Dice coefficient (DSC) was used to measure the quality of the segmentation, i.e., labels produced by the proposed method. The 5-fold cross validation was performed considering datasets split in a ratio of 70:10:20, i.e., training, validation, and testing.

We compared the results of the four models: (i) trained via plain self-training process, (ii) trained with the self-training process with incorporated mixup operation, (iii) baseline models without any further utilization of weak labels, and (iv) cross-consistency method proposed in Ouali et al. [32]. The first series of experiments demonstrates the impact of overall segmentation performance on the source dataset, while the target dataset is used to augment the source dataset via the self-training process. To check the segmentation performance, separate test data from the source dataset were used. The second set of experiments was conducted in which the model was trained in the same way as in the previous scenario, however, it was evaluated on data from a target dataset. In this context, we conducted experiments to cross inference between DFUC 2022 and FUSeg datasets to obtain comprehensive insights into the model’s generalization capabilities. In the final set of experiments, the RMIT dataset was employed to assess the segmentation performance of the proposed approach. However, it is important to note that this dataset contains only a limited number of ground truth labels, which have not been validated by medical specialists. Despite this limitation, we believe the results are noteworthy and have therefore chosen to report them separately.

The performance results when the training and test data belonged to the same dataset are shown in the “Source Domain Experiments” section, and domain adaptation results, i.e., when the two datasets were different, are in the “Domain Adaptation Experiments section.”

### Implementation Details

The first step was that all images were cropped into smaller sets of patches with an ROI size of 256x256 based on the label location to preserve the presence of an ulcer in each patch. In the next step, we scaled and normalized pixels to the interval [0, 1]. Additionally, three augmentation transforms were incorporated into the training pipeline: flipping across both axes, random rotation within 25 degrees range, and random zooming with maximum zoom set to two.

Attention U-Net [33] architecture, which was previously successfully applied in other segmentation scenarios of pathological structures [34–36] was used as a baseline model in this study. Each Attention U-Net was trained for 750 epochs using the AdamW optimizer, with the initial learning rate set to 0.001. The batch size was set to eight, with mixup augmentation parameter $\lambda \sim \beta (\alpha = 0.2)$. As a loss function, we used a combination of Dice and cross-entropy functions. During the inference process, we utilized the sliding window technique with an ROI size of 256x256. By systematically analyzing regions of interest at this fixed size, our approach maintained consistency and accuracy in identifying ulcerous regions while efficiently managing computational resources. The whole training process was performed on one Nvidia RTX 3060 GPU with 12 GB of computing memory. On average, it took about 15 h to train one model.

### Source Domain Experiments

**Fig. 3:**
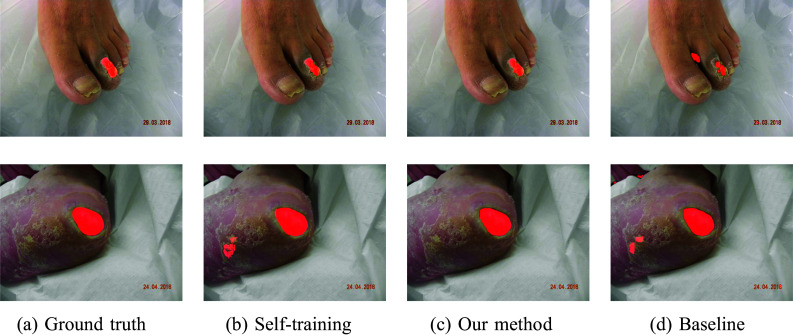
Example segmentations from the DFUC 2022 dataset in source domain experiments

**Fig. 4:**
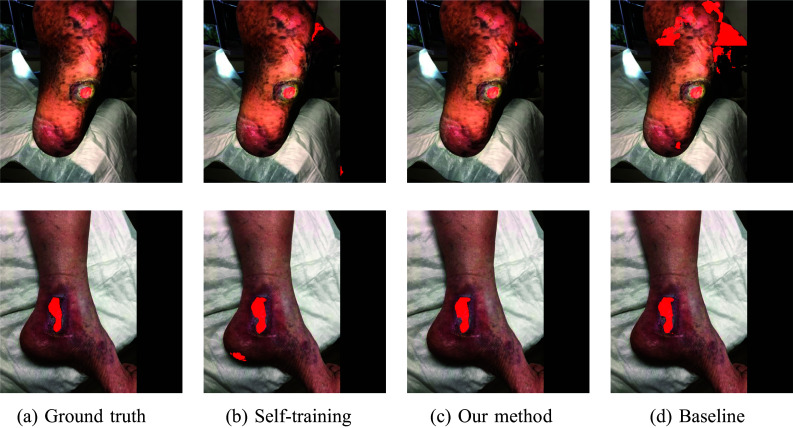
Example segmentations from the FuSeg dataset in source domain experiments

To determine the performance when the training and test data belonged to the same dataset, the experiments were repeated for the two datasets independently and are shown in Table 1. Visualization of selected samples from both datasets and their segmentations are shown in Figs. 3 and 4. Our approach notably enhances network segmentation precision across both datasets. This can be observed in the presented samples, where our method demonstrates a tendency to disregard background noise, distinguishing it from other methodologies. The performance of self-training with the combination of mixup surpassed the unmodified baseline model measured by DSC. Additional application of mixup to self-training turned out to produce better results than the plain self-training method, which was itself at the same level of accuracy as the baseline model.

**Table 1:** Segmentation performance in source domain experiments measured by DSC

Source	Target	Self-training	Ours	Baseline	CCT
DFUC 2022	DFUC 2022	$0.689 \pm 0.04$	$\mathbf {0.711 \pm 0.043}$	$0.692 \pm 0.037$	$0.529 \pm 0.024$
FuSeg	FuSeg	$0.841 \pm 0.007$	$\mathbf {0.859 \pm 0.01}$	$0.814 \pm 0.03$	$0.621 \pm 0.014$

Bolded text marks the best obtained results

### Domain Adaptation Experiments

In the second set of experiments, we focused on the evaluation of the domain adaptation capabilities of our proposed method. Table 2 shows the results and it can be observed that the combination of self-training and mixup augmentation performed better than both, the baseline method and plain self-training. In this scenario, the improvement appears to be more than seen in the “Source Domain Experiments” section, where the model and test data belonged to the same dataset. This shows that the baseline method and plain self-training performed worse during the generalisability test, while our model was more robust.

**Table 2:** Segmentation performance in domain adaptation experiments measured by DSC

Source	Target	Self-training	Ours	Baseline	CCT
FUSeg	DFUC 2022	$0.523 \pm 0.047$	$\mathbf {0.561 \pm 0.044}$	$0.541 \pm 0.031$	$0.432 \pm 0.024$
DFUC 2022	FUSeg	$0.69 \pm 0.03$	$\mathbf {0.714 \pm 0.016}$	$0.677 \pm 0.037$	$0.461 \pm 0.029$

Bolded text marks the best obtained results

**Fig. 5:**
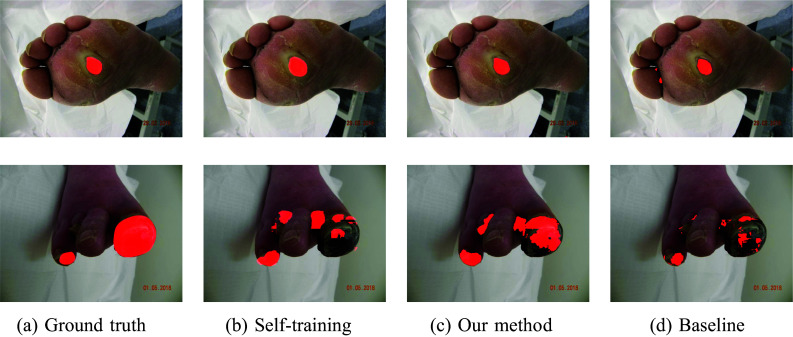
Example segmentations from the DFUC 2022 dataset in domain adaptation experiments

**Fig. 6:**
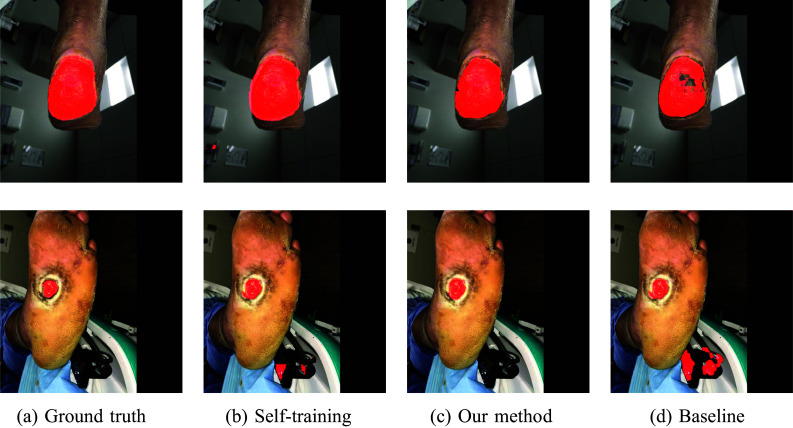
Example segmentations from the FuSeg dataset in domain adaptation experiments

The bigger difference in terms of DSC and improved reliability in predicting ulcer location were observed when the DFUC 2022 dataset was used as a source and we argue that this phenomenon can be caused by the fact that DFUC 2022 contains more samples than FUseg, the model was able to better capture ulcers attributes. The pictures of selected samples from both datasets and their segmentations are shown in Figs. 5 and 6.

### Validation on RMIT Dataset

The new proposed segmentation approach was evaluated in real-life conditions using the RMIT dataset. We would like to stress that the RMIT dataset has not been annotated yet by a specialist. Therefore, we provide only several examples of interference on this dataset. The ground truth masks for these samples were prepared by one of the authors before he saw actual predictions.

**Fig. 7:**
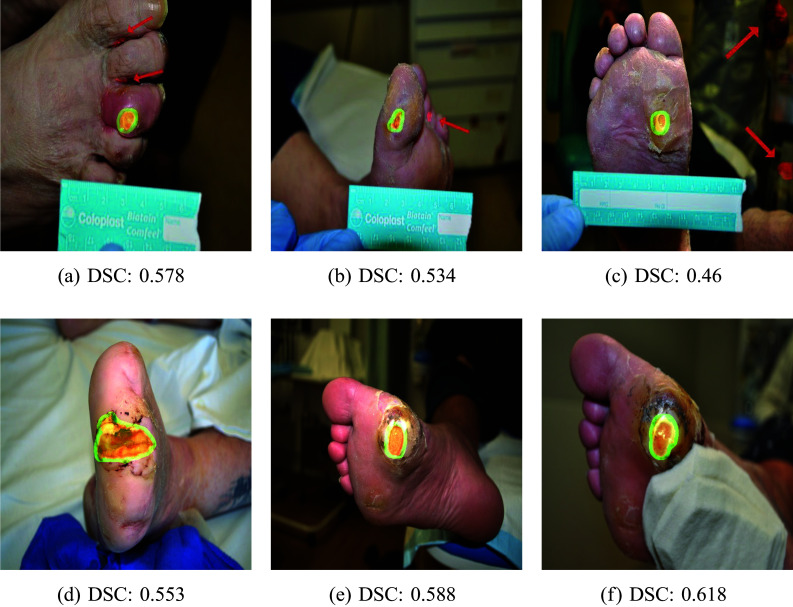
Samples from RMIT dataset. Red color marks model prediction, green marks human annotation, and orange represents an overlay of these predictions

The source dataset for initial model training in this case was the DFU2022 dataset. Images and annotations were evaluated using the DSC metric, similar to previously presented results. Samples of images together with metric values can be seen in Fig. 7. The visual inspections of the samples suggest that the segmentation performed by our model was close to that done manually. It was also observed that the location was correctly identified. The average DSC was equal to **0**.**555**.

Visual inspection of the segmentation, such as in Fig. 7a and b shows that the model has the tendency to over-estimate the ulcer, which may be due to factors such as skin texture around the ulcer being identified as the ulcer. Sample 7c also shows that the model can identify the ulcer from the region corresponding to the background, i.e., outside the foot area. On visual inspection, we observed that this was when the background was noisy. Two, yet untested approaches to overcome this are (i) cropping of the image and (ii) adding a layer into the framework to firstly segment the foot area to remove the background.

## Discussion

In this study, we have found that by adding mixup augmentation to the self-training process improves the segmentation performance of foot ulcers. The dice coefficient increased when training and testing the proposed model using the FUSeg dataset. The results were similar to those produced to the ones presented in the original challenge paper [30]. We believe that our method could produce better results with further fine-tuning.

When the mixup model was trained on one dataset and tested on another dataset, the segmentation precision was better than the baseline Attention U-net model [33]. To further verify the adaptation capabilities of our proposed method, we evaluated the trained DFUC 2022 model from the “Domain Adaptation Experiments” section on unseen samples from the RMIT dataset to generate diabetic foot ulcer predictions and compared these with an untrained human annotator.

This paper has presented the incorporation of mixup augmentation in the training process designed by the self-training method to effectively utilize datasets, where labels are missing. Lack of label presence is a common scenario in the context of image analysis, especially in medical image segmentation, and any method to overcome this limitation can help enhance the applicatgion of AI for medical image analysis. While our approach is promising, it is essential to test other methods with mixup augmentation techniques for other applications. It is also important to test other augmentation methods which may provide alternate methods. One option is to implement automated augmentation techniques such as AutoAugment [37] to find the optimal set of augmentation operations and apply them later during the self-training process.

Another approach could be the utilization of different regularization methods such as label smoothing to improve generalization and adaptation capabilities. One can generate soft labels for targeted classes to introduce a level of uncertainty during training and modify training loss to ensure that the model is penalized not only for incorrectly classified pixels but also for being overly confident in predictions.

An one aspect of our proposed domain adaptation approach is its theoretical applicability across various network architectures. While our experiments have demonstrated improvements using the U-Net architecture, the underlying principles of our method are not restricted to this model alone. The techniques of generating weak labels through self-training and enhancing dataset robustness with mixup augmentation can theoretically be integrated with the most of the neural network architectures. This flexibility suggests that the method could be adapted for use with more recent or advanced neural network models, potentially enhancing performance or efficiency in other complex segmentation tasks beyond diabetic foot ulcer segmentation.

Overall, self-training is a promising approach and can be modified and enhanced in many different ways, which can contribute to deep learning training in the medical domain and we are encouraged to further explore this topic. This study has still some limitations which need to be addressed for it to be effective for use in the real-world applications. The first is that experiments were performed on limited data size and corresponding to the plantar foot images, specific application. The scope of segmentation was narrowed down to the segmentation of the foot ulcers. Thus, this method cannot be claimed to be suitable for segmenting medical images in general because each application may have its own unique challenges.

## Conclusion

This paper has presented a novel approach to improve domain adaptation in diabetic foot ulcer segmentation. It was evaluated on three datasets of the DFU images. The results confirm that segmentation of diabetic foot ulcers in unseen domains can be improved by using self-learned weak labels together with mixup augmentation and source data. This approach was able to overcome the baseline model approach limitation and it performed better than both, the baseline and the self-training method.

We believe that our framework can be further extended and improved. Possible modifications include replacing mixup augmentation with other variants and experimenting with other augmentation techniques. One can further exploit the self-training method and adjust the mechanism to incorporate the ensemble technique or use other generative methods to produce weak labels from unseen domains.

The outcomes of this study are positive and can lead to many diverse potential applications, which however are out of the scope of this paper. In our future work, we intend to include a number of diverse scenarios, datasets, and domains.

## Data Availability

FUSeg dataset is openly available at https://github.com/uwm-bigdata/wound-segmentation/blob/master/data/Foot%20Ulcer%20Segmentation%20Challenge/README.MD. DFUC 2022 dataset can be obtained at https://dfuc2022.grand-challenge.org/. RMIT dataset is not publicly available due to legal restrictions.
